# The therapeutic effect of IPL on children with vernal keratoconjunctivitis

**DOI:** 10.3389/fmed.2026.1853654

**Published:** 2026-06-22

**Authors:** Jiao Jiang, Shaoyi Wang, Shaohua Chen, Shuoshuo Meng, Yang Yang

**Affiliations:** 1 Department of Ophthalmology, Hebei Children's Hospital, Shijiazhuang, Hebei, China; 2 Department of Ophthalmology, Hebei Clinical Medicine Research Center for Children's Health and Diseases, Shijiazhuang, Hebei, China; 3 Department of Abdominal Ultrasonography, The Second Hospital of Hebei Medical University, Shijiazhuang, Hebei, China

**Keywords:** allergic conjunctivitis, intense pulsed light, meibomian gland function, ocular surface symptom questionnaire, vernal keratoconjunctivitis

## Abstract

**Objective:**

To observe the effectiveness of intense pulsed light (IPL) treatment as an adjuvant treatment of vernal keratoconjunctivitis (VKC) in children.

**Methods:**

Sixty-two children diagnosed with VKC from January 2022 to November 2024 were selected and divided into the IPL group (27 cases, conventional drug therapy combined with IPL treatment) and the control group (35 cases, conventional drug therapy). Symptom and sign indicators were tested before treatment (baseline) and after the end of treatment. The Treatment course, recurrence time and the number of adverse reactions for each group of patients were recorded.

**Result:**

There was no statistically significant difference in the treatment course between the two groups (*P* > 0.05); IPL treatment independently and significantly extended the recurrence interval in children with VKC (log-rank *P* = 0.003). There were no adverse reaction in either group during the treatment.

**Conclusion:**

IPL can effectively prolong the recurrence interval of VKC, and has good safety.

## 1 Introduction

Vernal keratoconjunctivitis (VKC) (1) is a chronic ocular inflammatory disease with a tendency for seasonal recurrence, involving the cornea and conjunctiva. It commonly occurs in children and adolescents, especially those under the age of ten. The main symptoms and signs include eye itching, foreign body sensation, increased secretions, conjunctival congestion, edema, palpebral conjunctival papillae hyperplasia, and Horner-Trantas nodules at the corneal limbus. The pathogenesis of VKC may involve genetics, endocrine factors, immune mediation, and environmental factors. Currently, the clinical treatment mainly consists of eye drops such as antihistamines, mast cell stabilizers, glucocorticoids, and immunosuppressants, with long treatment courses and inability to fundamentally prevent disease recurrence (2). Intense Pulsed Light (IPL) is a broad-spectrum non-coherent light. In recent years, it has gradually been applied in the field of ophthalmology for the treatment of ocular surface diseases. Its mechanism of action mainly includes closing abnormal dilated blood vessels in the conjunctiva, controlling the activation of inflammatory factors on the ocular surface, thereby inhibiting ocular surface inflammatory responses and improving meibomian gland function, etc. Our team previously applied IPL as an adjunctive treatment for refractory allergic conjunctivitis in children, showing good efficacy (3). Studie have shown (4) that IPL can alleviate oxidative stress reactions in perennial allergic conjunctivitis. Based on this, for patients with frequently recurring VKC, our research has attempted to use IPL as an adjunctive treatment for VKC and to observe its efficacy.

## 2 Object and methods

### 2.1 Research object and group

The study subjects were selected from patients diagnosed with VKC at the Ophthalmology Department of Hebei Children's Hospital from January 2022 to November 2024. The patients had to have experienced at least three recurrences within the past 1 year. A total of 62 patients were included. Among them, 27 patients (IPL group) agreed and accepted conventional drug therapy combined with IPL treatment, and 35 patients (control group) received conventional drug therapy. Both groups selected the right eye as the research subject.

#### 2.1.1 Inclusion criteria

(1) Children under the age of 15, with good compliance, and able to cooperate with all examinations and follow-ups; (2) Meeting the diagnostic criteria for VKC (5); (3) In the period of illness onset, with obvious subjective symptoms (itching, foreign body sensation, etc.); (4) Recurrent at least three times within the past 1 year; (5) No history of wearing contact lenses; (6) No other inflammatory diseases or conditions that may cause dry eye or meibomian gland dysfunction; (7) Normal intraocular pressure; (8) No autoimmune diseases.

#### 2.1.2 Exclusion criteria

(1) Within the past 1 month, have used immunosuppressants or glucocorticoids systemically; (2) Have a history of eye surgery or eyelid deformity; (3) Have a photosensitive disorder.

As this was a retrospective study, all eligible patients from the study period were included without *a priori* sample size calculation; the unequal group sizes reflect real-world patient preference for adjunctive IPL treatment.

Our study was approved by the Ethics Committee of Hebei Children's Hospital, which complies with the requirements of the Helsinki Declaration. The parents of the children were informed of and agreed to participate in this study, and signed the informed consent form. This study was conducted in accordance with the STROBE guidelines. The completed STROBE checklist is provided as Supplementary material.

### 2.2 Treatment methods

#### 2.2.1 Control group

Received conventional drug treatment. Eye drops included antihistamine drugs, mast cell stabilizers, glucocorticoids, immunosuppressants and artificial tears. According to the severity of the disease (Bonini scale), the drug treatment plan is as follows: mild-moderate (Grade 1–2): olopatadine hydrochloride 0.1% eye drops, 2 × /day; sodium hyaluronate 0.3% artificial tears, 4 × /day. Moderate-severe (Grade 3–4): above plus fluorometholone 0.1% eye drops, 2 × /day ( ≤ 2 weeks); cyclosporine A 0.05% emulsion, 2 × /day. All drugs used were of the same brand and dosage form in both groups.

#### 2.2.2 IPL group

Conventional drug treatment combined with IPL treatment. For the IPL treatment of patients, the “EYESIS Light Pulse MOPT Meibomian Gland Dysfunction Treatment Instrument” was used. IPL energy was started at 8 J/cm^2^ and increased stepwise by 0.5 J/cm^2^ up to a maximum of 12 J/cm^2^ based on the child's tolerance (no pain or mild warmth). The same physician (J. J.) performed all treatments to ensure consistency. The children were in a supine position, both eyes were covered with protective eye masks, and coupling agent was applied to the lower eyelid. The same physician held the treatment handle and used the fan-shaped irradiation method to perform IPL treatment from the lower eyelid nasal side to the temporal side. Each eye was irradiated five times. IPL treatment was received once a week for a total of three times.

### 2.3 Observation indicators

All patients were followed for a minimum of 12 months after recovery, with regular visits every 4 weeks. Visual acuity, intraocular pressure, slit-lamp examination and corneal fluorescein staining examination were performed at each follow-up visit.

#### 2.3.1 Symptoms and signs

Symptom and sign indicators were tested before treatment (baseline) and after the end of treatment. Ocular surface symptoms (Supplementary Figure S1): filled out by the guardian and the child together, and the total score was recorded. Ocular surface signs (Supplementary Figure S2): referencing the Bonini scale (6), the same physician conducted the examination of the patient under a slit lamp and recorded the grading. The treatment endpoint (recovery) was defined as a symptom score of 0 and a sign grading of 0.

#### 2.3.2 Treatment course

The number of weeks from the first diagnosis to the achievement of symptom score 0 and sign grade 0 (clinical recovery).

#### 2.3.3 Recurrence time

The number of weeks from the date of clinical recovery to the date when the patient again presented with typical VKC symptoms (itching, congestion, papillae). That is, typical symptoms of viral keratitis (itching, redness) occur and the severity level is ≥1.

#### 2.3.4 Safety

Record the number of cases of adverse reactions such as eye swelling, pain, and increased intraocular pressure during the treatment period and the follow-up period.

### 2.4 Statistical analysis

The analysis was conducted using the statistical software IBM SPSS Statistics26. Normality of continuous variables was assessed using the Shapiro–Wilk test. All continuous variables (age, ocular surface symptoms score, ocular surface signs grade, treatment course, and recurrence time) were normally distributed. Age, ocular surface symptoms score, ocular surface signs grade and treatment course are expressed as mean ± standard deviation (x ± s), and comparisons between groups were performed using the independent sample t-test. Performed multivariate linear regression to adjust for potential confounders including age, gender, baseline ocular surface symptom score, and baseline ocular surface sign grade. The recurrence time was estimated using the Kaplan-Meier method, and the comparison between the two groups was conducted using the Log-Rank test. The gender were expressed as rates (%), and the comparison between groups was performed using the Pearson χ^2^ test. *P* < 0.05 indicated statistically significant differences. Normality of continuous variables was assessed using the Shapiro–Wilk test. All continuous variables (age, ocular surface symptoms score, ocular surface signs grade, treatment course, and recurrence time) were normally distributed (*P* > 0.05 for all).

## 3 Results

### 3.1 Baseline data

There was no statistically significant difference in age, gender, ocular surface symptoms score and ocular surface signs grade among IPL group and control group (*P* > 0.05), so they were comparable (Table 1).

**Table 1:** Comparison of age, gender, ocular surface symptoms, ocular surface signs differences between IPL group and control groups.

Indicator	Group		*t*	*p*
	IPL group (*n* = 27)	Control group (*n* = 35)		
Ocular surface symptoms	8.93 ± 3.16	9.97 ± 4.17	−1.123	0.266
Ocular surface signs	2.48 ± 0.64	2.20 ± 0.76	1.545	0.128
Age	7.70 ± 1.75	7.20 ± 1.78	1.113	0.270
Female	13 (48.15%)	14 (40.00%)	—	0.521
Male	14 (51.85%)	21 (60.00%)		

### 3.2 Treatment course

**Figure 1:**
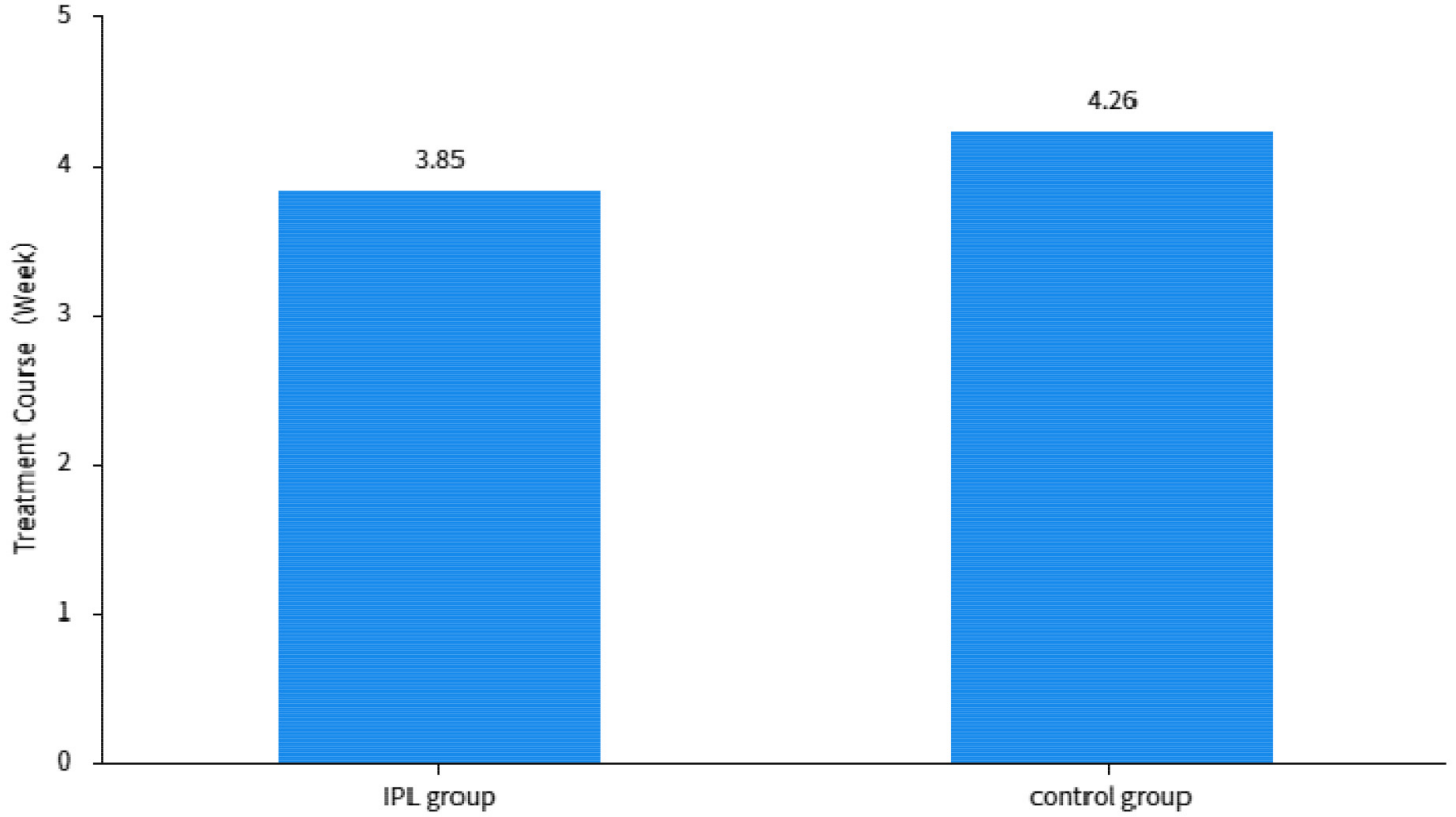
Comparison of treatment course between IPL group and control group.

The treatment course was 3.85 ± 0.91 weeks for the IPL group and 4.26 ± 1.15 weeks for the control group, the difference was not statistically significant (*P* > 0.05; Table 2, Figure 1).

**Table 2:** Comparison of treatment course between IPL group and control group.

Indicator	Group		*t*	*p*
	IPL group (*n* = 27)	Control group (*n* = 35)		
Treatment course	3.85 ± 0.91	4.26 ± 1.15	−1.508	0.137

### 3.3 Recurrence time

**Figure 2:**
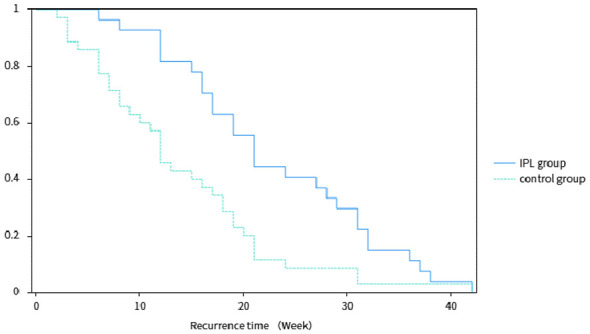
The survival curve of the IPL group was consistently above that of the control group, and the median recurrence time was significantly longer than that of the control group.

The median recurrence time was 21.00 weeks (95% CI: 16.00–29.00) in the IPL group and 12.00 weeks (95% CI: 8.00–17.00) in the control group. The log-rank test indicated a significant difference between the two groups (*P* = 0.003; Table 3, Figure 2).

**Table 3:** Comparison of recurrence time between IPL group and control group.

Indicator	Group	Median	95% CI	χ^2^	*p*
Recurrence time	IPL group	21.00	16.000–29.000	8.976	0.003
	Control group	12.00	8.000–17.000		

**Figure 3:**
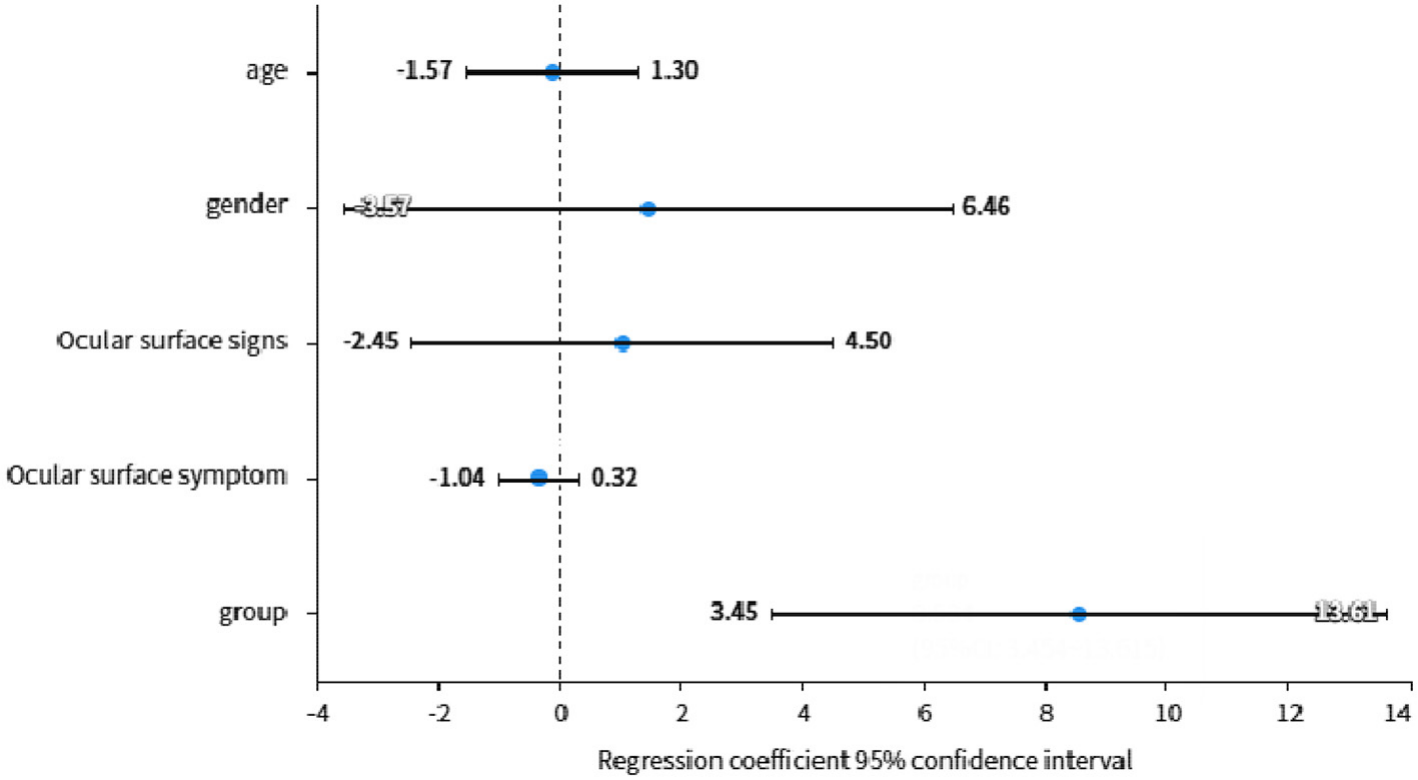
Forest plot of multivariate linear regression for recurrence time. Only the IPL treatment group (group) had a 95% CI that did not cross the null line at 0, indicates that it is an independent influencing factor for the prolongation of recurrence time.

Multivariate linear regression demonstrated that IPL treatment was independently associated with a longer recurrence time (95% CI: 3.45–13.61, *P* = 0.003). Age, gender, baseline symptom score, and baseline sign grade had no significant effects on recurrence time (all *P* > 0.05; Figure 3).

## 4 Discussion

The recurrent and persistent nature of VKC can lead to severe complications such as corneal neovascularization, scarring, keratoconus, and even steroid-induced glaucoma (7). Recent transcriptome profiling of conjunctival epithelial cells from VKC patients has provided molecular evidence of significant epithelial remodeling, although this remodeling does not appear to be mediated by epithelial-mesenchymal transition (8). The commonly used clinical treatment plans for VKC are eye drops as follows: mild cases are treated with histamine drugs/histamine receptor stabilizers, moderate to severe cases require the combination of glucocorticoids, and refractory or steroid-dependent cases need to add immunomodulators such as cyclosporine or tacrolimus (9). However, even with the above standardized treatment, VKC still has a significant tendency to recur, and some children even present with a perennial course of the disease (10). In our study, the average recurrence interval of the control group was 16.11 weeks, further suggesting that relying solely on conventional drugs is insufficient to ensure the long-term stability of the condition.

Our study found that the first recurrence interval for patients in the IPL combined treatment group was significantly longer than that of the drug-only group, suggesting that IPL can effectively prolong the remission period. The mechanism of action may be related to the following pathways: firstly, IPL can close abnormal dilated capillaries in the conjunctiva and the ocular surface through selective photothermal action (3). In VKC patients, there are a large number of new blood vessels and inflammatory cells (eosinophils, mast cells, lymphocytes) infiltrating the conjunctiva tissue, and these cells are the key cellular basis for the persistence and recurrence of inflammation (11). Secondly, IPL can inhibit the activation and release of various inflammatory factors in the ocular surface. A large number of studies have shown that the levels of Th2-type cytokines (IL-4, IL-5, IL-13), chemokines (CCL24, CCL11, CCL17), and eosinophil cationic protein (ECP) in the tear fluid of VKC patients are significantly increased (12, 13). In addition, Li et al. (4) found that IPL can significantly alleviate the oxidative stress response in perennial allergic conjunctivitis, and oxidative stress has been confirmed to be involved in the chronic inflammatory process of VKC (14). The follow-up duration is relatively short, limiting our ability to assess long-term recurrence patterns, steroid-sparing effects, or delayed adverse events such as effects on corneal topography or intraocular pressure beyond one year. We are continuing to follow this cohort for longer-term outcomes. Based on previous studies in allergic conjunctivitis and meibomian gland dysfunction, the beneficial effects of IPL may be mediated by several pathways, including closure of abnormal conjunctival vessels, inhibition of inflammatory cytokines, and reduction of oxidative stress. However, our study did not directly measure these parameters, and these mechanisms remain speculative in the context of VKC. Future mechanistic studies measuring tear cytokines and ocular surface oxidative stress markers are needed.

In terms of the treatment course, there was no significant difference between the two groups of patients. The pathogenesis of VKC includes IgE-mediated immediate allergic reactions, as well as chronic inflammation dominated by Th2 cells, imbalance of oxidative stress, and damage to the ocular surface barrier function (2, 14). Conventional anti-allergic drugs can only relieve the allergic symptoms during the acute phase and cannot repair the ocular surface barrier damage, improve abnormal tear film, and restore the homeostasis of the ocular surface microenvironment. This is also an important reason why the recurrence rate is relatively high after simple drug treatment (5, 15). However, conventional antihistamine drugs and glucocorticoids still have an irreplaceable advantage in rapidly controlling acute inflammation. Therefore, intense pulsed light is more suitable as an auxiliary treatment method after the acute symptoms have been effectively controlled, to reduce the chronic inflammatory load of the ocular surface, and thereby extend the interval between disease recurrences.

In our study, no adverse reactions such as elevated intraocular pressure, corneal damage, or pain were observed in all cases during the treatment and follow-up periods. This is consistent with the previous research conclusions on the application of IPL in meibomian gland dysfunction (16, 17), indicates that low-energy and short-course IPL treatment may be safe and feasible. However, given the relatively small sample size and limited follow-up, long-term safety requires further confirmation in larger studies.

Our study has certain limitations: the most important limitations of this study are its retrospective, non-randomized design and the allocation of patients based on guardians' preference, which may have introduced selection bias. As noted in the TFOS DEWS II report, many studies on ocular surface treatments face similar challenges in randomization, blinding, and sample size, and our limitations reflect the general state of evidence in this field (18). Therefore, our results should be interpreted as hypothesis-generating rather than confirmatory; Another limitation is the absence of objective biomarkers such as tear cytokines, meibography, tear break-up time, or tear osmolarity. Subjective symptom and sign scores, although clinically relevant, are prone to observer and recall bias. Future prospective studies should include quantitative objective measures to enhance reproducibility and mechanistic interpretation. A well-designed prospective randomized controlled trial is warranted to establish causal inference. And the relatively small and uneven sample size may limit statistical power for secondary outcomes and safety analyses. Future studies should perform sample size calculations to ensure adequate power.

## 5 Conclusions

In conclusion, the combination of IPL and conventional drugs in the treatment of childhood VKC can significantly prolong the recurrence interval, with good safety. IPL achieves this through multiple mechanisms such as closing abnormal blood vessels, inhibiting inflammatory factors, reducing oxidative stress, and improving the microenvironment of the ocular surface. These mechanisms compensate for the shortcomings of traditional drug treatment, which can only alleviate acute symptoms but is unable to block chronic inflammation and disease recurrence. Therefore, IPL is suitable as an adjuvant therapy method for children with recurrent VKC.

## Data Availability

The raw data supporting the conclusions of this article will be made available by the authors, without undue reservation.
