# Supplementary figures and images for: The therapeutic effect of IPL on children with vernal keratoconjunctivitis
Source: Front Med (Lausanne). 2026 Jun 22;13:1853654. doi: 10.3389/fmed.2026.1853654 (PMC13333480; doi:10.3389/fmed.2026.1853654)

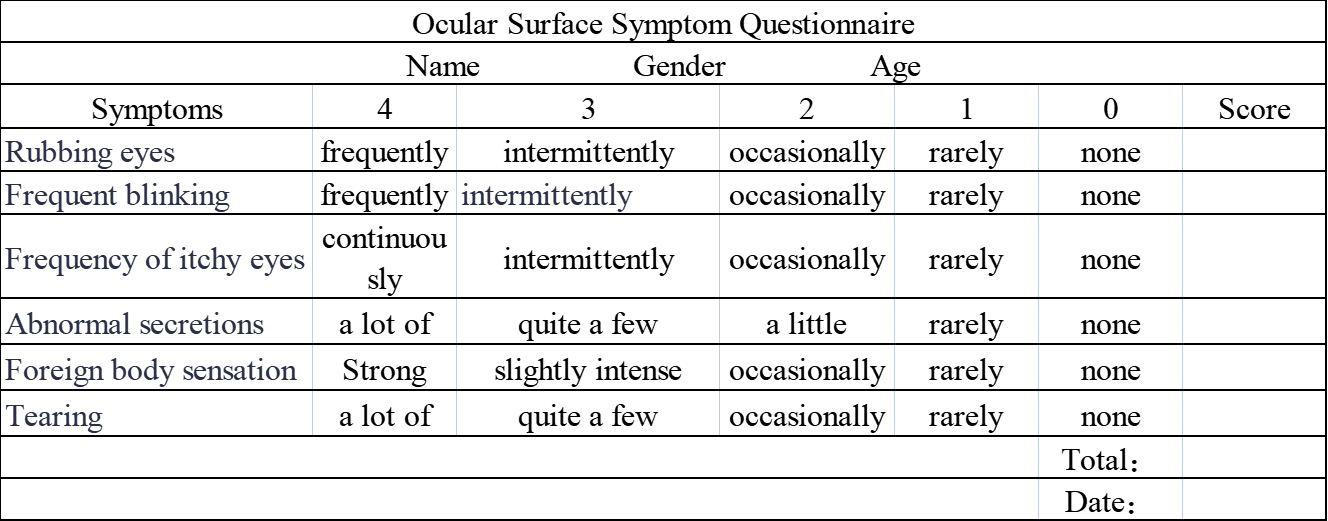


Supplementary Figure S1 Ocular Surface Symptoms


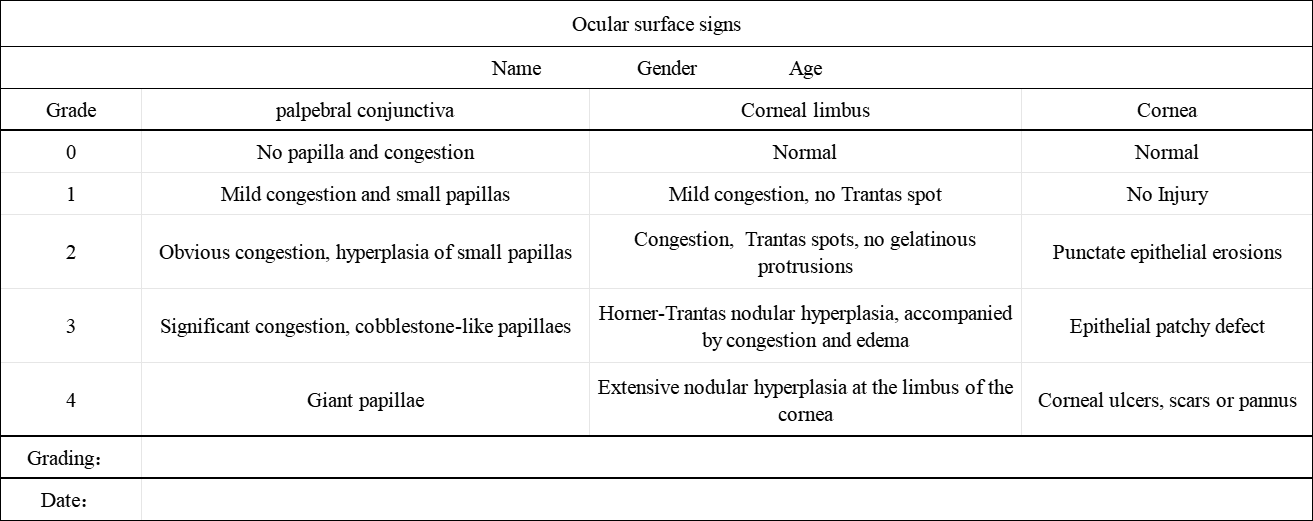


Supplementary Figure S2 Ocular Surface Signs

Supplement: Supplementary file 1 [file Table_1.doc]
